# Interleukin-18 Is a Potential Biomarker Linking Dietary Fatty Acid Quality and Insulin Resistance: Results from a Cross-Sectional Study in Northern Italy

**DOI:** 10.3390/nu15071782

**Published:** 2023-04-06

**Authors:** Domenico Sergi, Juana Maria Sanz, Stefano Lazzer, Gloria Brombo, Giovanni Zuliani, Gianni Biolo, Boštjan Šimunič, Rado Pišot, Edoardo Dalla Nora, Angelina Passaro

**Affiliations:** 1 Department of Translational Medicine, University of Ferrara, Via Luigi Borsari, 46, I-44121 Ferrara, Italy; 2 Department of Chemical and Pharmaceutical Sciences, University of Ferrara, Via Luigi Borsari, 46, I-44121 Ferrara, Italy; 3 Department of Medicine, University of Udine, Piazzale M. Kolbe 4, I-33100 Udine, Italy; 4 Medical Department, University Hospital of Ferrara Arcispedale Sant’Anna, Via A. Moro 8, I-44124 Ferrara, Italy; 5 Department of Medicine, Surgery and Health Sciences, University of Trieste, Strada di Fiume, 447, I-34149 Trieste, Italy; 6 Institute for Kinesiology Research, Science and Research Centre of Koper, Garibaldijeva 1, SI-6000 Koper, Slovenia

**Keywords:** IL-18, metabolic inflammation, insulin resistance, saturated fatty acids, monounsaturated fatty acids, omega-3 fatty acids, polyunsaturated fatty acids

## Abstract

Dietary lipids are pivotal in modulating metabolic inflammation. Among the inflammatory mediators characterizing metabolic inflammation, interleukin 18 (IL-18) has been consistently associated with obesity and insulin resistance. This study aims to evaluate whether the quality of lipid intake impacts upon IL-18 plasma levels and the implications on insulin resistance computed by the homeostatic model assessment for insulin resistance (HOMA-IR). Using a cross-sectional design, this study confirmed that IL-18 correlated positively with insulin resistance and individuals with a HOMA-IR ≥ 2.5 displayed higher circulating IL-18 levels compared with their insulin-sensitive counterparts. In terms of the effect of the quality of dietary lipids on IL-18 circulating levels, the ratio between monounsaturated, omega-3, polyunsaturated and saturated fatty acids as well as the intake of eicosapentaenoic and docosahexaenoic acids correlated negatively with IL-18. Despite this, IL-18 circulating levels, but not dietary fatty acid quality, predicted insulin resistance. Nevertheless, the ratio between omega 3 and saturated fatty acids was a predictor of IL-18 plasma levels. Thus, the downregulation of IL-18 may underpin, at least partially, the beneficial metabolic effects of substituting omega 3 for saturated fatty acids with this cytokine potentially representing a biomarker linking dietary lipids and metabolic outcomes.

## 1. Introduction

Obesity has reached the proportion of an epidemic worldwide, therefore posing a huge burden to people’s health as well as to the healthcare systems in developed as well as developing countries [1]. Indeed, obesity represents a pivotal risk factor for a plethora of comorbidities including type 2 diabetes mellitus (T2DM), cardiovascular disease, several types of cancer and neurodegenerative diseases [2,3,4].

Obesity is characterised by a state of low-grade chronic inflammation, termed metabolic inflammation, which is primarily driven by hypertrophic adipocytes in concert with the immune cells, mainly macrophages, infiltrating the dysfunctional adipose tissue of obese individuals [5]. Indeed, adipocyte expansion to accommodate the accumulation of excess energy and macrophage polarisation towards an M1 phenotype are paralleled by an increase in the secretion of pro-inflammatory mediators from the adipose tissue. In particular, there is an increase in the secretion of pro-inflammatory cytokines such as TNFα, IL-6 and IL-1β and a concomitant decrease in the release of anti-inflammatory, insulin-sensitising adipokines such as adiponectin [6].

The resulting low-grade chronic inflammation represents one of the underlaying factors linking obesity and insulin resistance [7,8,9,10]. As such, the activation of intracellular pro-inflammatory pathways such as the nuclear factor kappa-light-chain-enhancer of activated B cells (NF-κB) and the c-Jun *N*-terminal kinase (JNK) pathways can directly interfere with and impede insulin signalling by promoting insulin receptor (IRS) serine phosphorylation in metabolically active tissues, namely the skeletal muscle, adipose tissue and liver [11].

Besides the aforementioned pro-inflammatory mediators, obesity is characterised by an increase in the circulating levels of the pro-inflammatory cytokine IL-18, as well as its upregulation in the adipose tissue [12]. Remarkably, this pro-inflammatory cytokine has been widely associated with insulin resistance, both in terms of its circulating levels as well as its expression in the adipose tissue [12,13,14,15]. Additionally, IL-18 has been shown to be upregulated in individuals with the metabolic syndrome [16], with its circulating levels raising as the components of the syndrome increase [14]. Not surprisingly, IL-18 is also associated with hypertension [17] and dyslipidaemia [18], which, along with its ability to predict cardiovascular mortality [19], further supports the relationship between this cytokine and cardiometabolic health. Finally, besides being associated with insulin resistance, IL-18 may also contribute to its pathogenesis [20].

Diet is a key discriminant in shaping the low-grade chronic inflammatory status that characterises obesity, with healthy dietary patters such as the Mediterranean diet inhibiting [21,22] and unhealthy dietary regimens such as the Western diet fostering inflammation [23]. The same holds true for IL-18, with a Mediterranean-like diet, emphasising the consumption of fruit and vegetables while decreasing the intake of meat and fat from animal sources, decreasing circulating IL-18 levels [24]. In terms of the dietary components able to modulate metabolic inflammation, dietary lipids play a crucial role. Indeed, while long-chain saturated fatty acids have been widely associated with inflammation [10,25,26,27], monounsaturated fatty acids and polyunsaturated fatty acids elicit anti-inflammatory responses [28]. To the same extend, while the former group of fatty acids has been linked with insulin resistance, the latter have been reported to improve insulin sensitivity [29,30]. Therefore, the balance between dietary saturated and unsaturated fatty acids is key in shaping cardiometabolic health. In this regard, omega-3 supplementation has been reported to lower IL-18 circulating levels [31]. However, it remains to be fully elucidated how different dietary lipids and their ratios modulate this cytokine. Thus, the aim of this study was to investigate how saturated and unsaturated fatty acids and their ratios affect IL-18 circulating levels in a cohort of non-diabetic individuals and the repercussions on insulin resistance.

## 2. Subjects and Methods

### 2.1. Participants

Four-hundred and three participants aged 55–80 years from the PANGeA project (https://ec.europa.eu/regional_policy/en/projects/italy/pangea-keeping-an-ageing-population-moving) were considered for this study. Inclusion criteria were free-living individuals and able to walk for 2 km without any aids. Exclusion criteria were cancer diagnosis, a history of hospitalization in the last 12 months or the therapy with anticoagulants. PANGEeA’s participants with missing food frequency questionnaires or IL-18 measures were excluded. Study participants underwent anamnestic and nutritional interviews, anthropometric measurements and blood sampling [32].

### 2.2. Dietary Assessment

Study participants were interviewed by trained nutrition expert medical doctors to fill food frequency and 24 h recall questionnaires.

Mediterranean diet adherence was calculated based on the frequency of consumption of 13 foods/food groups (milk and dairy products; cereals and grain products; vegetables; legumes; fruit, olive oil; white meat; red and processed meat; fish; sweets and desserts; nuts and seeds; and wine) according to guidelines of the Mediterranean diet [33], as specified by Sanz et al., 2022 [32].

The 24 h recall method is a retrospective and quantitative method to gather information about foods and beverages consumed by the participants in the 24 h period prior to the interview. Results were the average of the two 24 h recalls executed by the same interviewer, the first one personally on the day of the visit and the second one after 2 months over the phone. Data were analysed using the nutrient analysis software Winfood^®^ PRO 3.3 (Medimatica Surl, Teramo, Italy) to obtain total energy and macro and micronutrient intake for each participant.

### 2.3. Biochemical Analysis

Serum or plasma were obtained from fasting blood samples centrifuged at 1600× *g* for 15 min at 4°. Samples were aliquoted and stored at −80 °C until use. 

High-sensitivity *C*-reactive protein (hsCPR) was assessed using the immune-turbidimetric kit CRP OSR6147 (Beckman Coulter, Brea, CA, USA). Total cholesterol, HDL cholesterol, triglycerides, glucose and insulin were assayed by standard enzymatic colorimetric methods. LDL cholesterol was calculated by the Friedewald’s formula [34]. Insulin resistance was assessed using the homeostasis model assessment index (HOMA-IR), which was computed as follows:

$$\text{HOMA-IR}\;\mathrm{index}=\frac{\mathrm{glucose}\left(\frac{\mathrm{mmol}}{L}\right)\cdot \mathrm{insulin}\left(\frac{\mathrm{mU}}{L}\right)}{22.5}$$

### 2.4. Metabolic Syndrome

Metabolic syndrome was defined according to the National Cholesterol Education Program-Adult Treatment Panel III (NCEP ATP III) and diagnosed in the presence of three or more of the following five criteria: (1) waist circumference ≥ 102 cm in men or ≥88 cm in women; (2) systolic blood pressure ≥ 130 mmHg or diastolic blood pressure ≥ 85 mmHg or taking antihypertensive medications; (3) fasting triglycerides level ≥ 150 mg/dL or taking antihyperlipidaemic medications; (4) fasting HDL cholesterol ≤ 40 mg/dL in men or ≤50 mg/dL in women or pharmacological treatment for low HDL cholesterol; and (5) fasting blood glucose ≥ 110 mg/dL or taking hypoglycaemic medications.

### 2.5. Statistical Analysis

Continuous variables were expressed as mean ± standard deviation (SD) and median (quartile 1–quartile 3) and analysed using Shapiro–Wilk tests to identify normally and non-normally distributed variables. In some analyses, IL-18 and HOMA-IR, not normally distributed, were log transformed, as specified in figure legends. One way ANOVA or Mann–Whitney tests were used to assess overall differences between groups for normally and non-normally distributed parameters, respectively. To assess the physiological relevance, Cohen’s d (d) effect size was also calculated. Pearson’s or Spearman’s correlation coefficients were used to test the association between IL-18 and the parameters of interest. Stepwise multiple regression analysis was performed to reveal the independent predictors of HOMA-IR and IL-18 circulating levels. Data analysis was performed using SPSS Statistics for Windows, version 26.0 (SPSS, Inc., Chicago, IL, USA) and *p* ≤ 0.05 was considered statistically significant.

## 3. Results

### 3.1. Study Participant Characteristics

The characteristics of the study participants are reported in Table 1 and included their blood pressure, anthropometric and metabolic characterisation, encompassing fasting blood glucose, insulin, HOMA-IR and circulating lipid profile.

### 3.2. Relationship between IL-18 Circulating Levels, Insulin Resistance, Metabolic Syndrome and Body Composition

**Figure 1:**
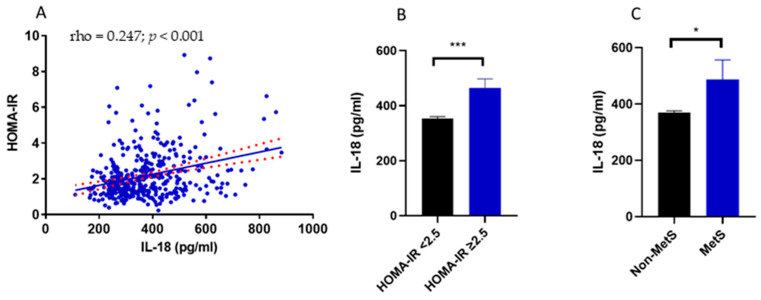
(**A**) Spearman correlation analysis between HOMA-IR and IL-18 circulating levels. Data analysis performed using Spearman correlation analysis is reported in Table 2. (**B**) IL-18 circulating levels in individuals with a HOMA-IR either <2.5 or ≥2.5. (**C**) IL-18 circulating levels in individuals affected by the metabolic syndrome or not meeting the diagnostic criteria for the metabolic syndrome. Data in B and C are reported as mean ± SEM. Differences between groups in B and C were assessed using *t*-test of log-transformed IL-18. * *p* value < 0.05, *** *p* value < 0.001. rho, Spearman’s rho correlation coefficient; HOMA-IR, homeostatic model assessment for insulin resistance; IL-18, interleukin 18; MetS, metabolic syndrome.

Considering the relationship between IL-18 and insulin resistance observed by other authors [12,13,14,15], we first aimed at elucidating whether this relationship held true in this study cohort. As expected, circulating IL-18 positively correlated with insulin resistance assessed by HOMA-IR (*p* < 0.001) (Table 2) (Figure 1A). In agreement with this, individuals with a HOMA-IR ≥ 2.5, set as the cut-off for insulin resistance [35], had higher IL-18 circulating levels compared with study participants with a HOMA-IR < 2.5 (*p* < 0.001 d = 0.641) (Figure 1B).

Moreover, in keeping with this, IL-18 circulating levels correlated positively with glucose and insulin levels (both *p* < 0.001). In terms of lipid profile, IL-18 plasma levels correlated positively with triglycerides (*p* < 0.05) and negatively with total (*p* < 0.01) and HDL-cholesterol (*p* < 0.001) (Table 2). However, insulin resistance also correlated with other anthropometric and metabolic parameters, as reported in Table 2.

Furthermore, considering the central role of insulin resistance in the pathogenesis of metabolic syndrome, it was next investigated whether IL-18 circulating levels differed between study participants affected by metabolic syndrome and their metabolically healthy counterparts. In line with the results relative to insulin resistance, the circulating levels of IL-18 were significantly higher in individuals with metabolic syndrome than their metabolically healthy counterparts (*p* < 0.05; d = 0.441) (Figure 1C).

To further confirm the relationship between insulin resistance and inflammation in the present cohort, high-sensitivity *C*-reactive protein (hsCRP) was assessed and its association with IL-18 and HOMA-IR was investigated. As expected, hsCRP correlated positively with both IL-18 circulating levels (*p* < 0.001) and HOMA-IR (*p* < 0.001).

Moreover, we identified a relationship between IL-18 and anthropometric parameters. In fact, IL-18 circulating levels correlated positively with BMI (*p* < 0.001), waist circumference (*p* < 0.001), hip circumference (*p* < 0.05), fat mass (*p* < 0.05), fat-free mass (*p* < 0.001) and muscle mass (*p* < 0.001) (Table 2).

### 3.3. Dietary Fatty Acids and Their Impact on IL-18 Circulating Levels

Dietary fatty acids are tightly linked to metabolic inflammation [36]. Thus, to confirm the impact of dietary lipids on inflammation, we investigated the relationship between their quality as well as quantity in the diet and IL-18 plasma levels. Despite total lipids, mono- and polyunsaturated fatty acid intake did not correlate with IL-18; this correlation tended to be positive for saturated fatty acids (*p* = 0.068) (Table 3).

On the contrary, the intake of EPA (*p* < 0.05), DHA (*p* < 0.01) and the ratios between monounsaturated/saturated fatty acids as well as omega-3/saturated fatty acids correlated negatively with IL-18 circulating levels (*p* < 0.01 and *p* < 0.001, respectively) (Table 3). Furthermore, this trend held true when considering the ratio between unsaturated fatty acid, both in terms of polyunsaturated fatty acids and the sum of mono- and polyunsaturated fatty acids and saturated fatty acid intake (*p* < 0.001) (Table 3). Finally, this effect was limited to dietary fatty acids, as no association was observed between cholesterol, carbohydrate, dietary fibre and protein intake, as well as alcohol consumption and IL-18 plasma levels (Table 3). Finally, total calories only showed a tendency to correlate positively with IL-18 plasma levels (*p* = 0.097) (Table 3).

Considering the Mediterranean diet being typically characterised by a high intake of mono- and polyunsaturated fatty acids and a low intake of saturated fatty acids, it was assessed whether IL-18 circulating levels were also associated with the adherence to this dietary pattern. In agreement with the data on dietary fatty acids, Mediterranean diet adherence score negatively correlated with plasma IL-18 levels (*p* < 0.05) (Table 3).

### 3.4. Dietary Fatty Acid Intake and Insulin Resistance

**Figure 2:**
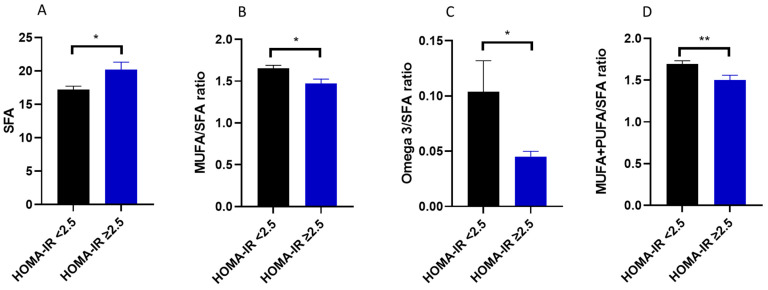
Dietary fatty acid quality consumed by individuals with a HOMA-IR either <2.5 or ≥2.5. (**A**) Intake of SFA, (**B**) ratio between MUFA and SFA, (**C**) ratio between omega-3 and SFA and (**D**) ratio between MUFA + PUFA and SFA. All data are expressed as mean ± SEM and differences between groups were assessed using Mann–Whitney test. * *p* < 0.05, ** *p* < 0.01. SFA, saturated fatty acids; MUFA, monounsaturated fatty acids; PUFA, polyunsaturated fatty acids; HOMA-IR, homeostatic model assessment for insulin resistance.

Considering the relationship between dietary fatty acid intake and IL-18 levels, it was next evaluated whether the quality of fatty acid intake was also related to insulin resistance. In keeping with this, HOMA-IR correlated negatively with the ratio between unsaturated (MUFA + PUFA)/saturated fatty acids (*p* < 0.01), monounsaturated/saturated fatty acids, omega 3/saturated fatty acids (*p* < 0.01) and the intake of DHA (*p* < 0.05) (Table 3). Instead, HOMA-IR correlated positively with the omega-6/omega-3 fatty acid ratio (*p* < 0.05) (Table 3). Furthermore, study participants with a HOMA-IR ≥ 2.5 also consumed more saturated fatty acids (*p* < 0.05; d = 0.245) (Figure 2A), a lower ratio of monounsaturated/saturated fatty acids (*p* < 0.05; d = 0.300) (Figure 2B), omega-3/saturated fatty acids (*p* < 0.05; d = 0.333) (Figure 2C) and unsaturated/saturated fatty acids (*p* < 0.01; d = 0.306) (Figure 2D).

Nevertheless, the intake of dietary fatty acids or their ratios were not identified as predictors of insulin resistance assessed by stepwise linear regression (Table 4). As expected, waist circumference was a predictor of and correlated positively with insulin resistance in model 1 (*p* < 0.001) (Table 4). Waist circumference prediction power was further enhanced by circulating triglycerides in model 2 (*p* < 0.01) (Table 4). Interestingly, IL-18 also correlated positively with insulin resistance and was able to predict HOMA-IR (*p* < 0.05), enhancing the prediction power of waist circumference and triglycerides in model 3 (Table 4). However, none of the other variables correlating positively with IL-18 circulating levels, including dietary fatty acids, were able to predict insulin resistance (Table 4).

### 3.5. Dietary Fatty Acid Quality as a Predictor of IL-18 Circulating Levels

The induction of metabolic inflammation represents a pivotal mechanism linking excess intake of long-chain saturated fatty acids and insulin resistance, with this effect being countered by monounsaturated and omega-3 fatty acids [29,30]. However, considering that dietary fatty acids were unable to directly predict insulin resistance, we investigated whether they represented predictors of IL-18 plasma levels. Alongside muscle mass, which proved to be a predictor of IL-18 in model 1 (*p* < 0.001), the omega-3/saturated fatty acid ratio improved the predictivity of muscle mass (*p* < 0.01) (model 2) on IL-18 circulating levels (Table 5). In model 3, the predictive power of muscle mass and omega-3/saturated fatty acid ratio on IL-18 plasma levels, albeit moderate, was further increased by the addiction of HDL-C (*p* < 0.05) and in model 4 by total cholesterol plasma levels (*p* < 0.05) (Table 5).

Although a partition of the study cohort was taking drugs that may affect lipid and carbohydrate metabolism, including anti-hypertensive and hypolipidemic drugs (Table 1), these agents did not affect the predictive power of muscle mass and omega-3/SFA ratio on IL-18 circulating levels (Table 5).

### 3.6. The Relationship between Muscle Mass and IL-18 Is Influenced by Fat Mass

**Figure 3:**
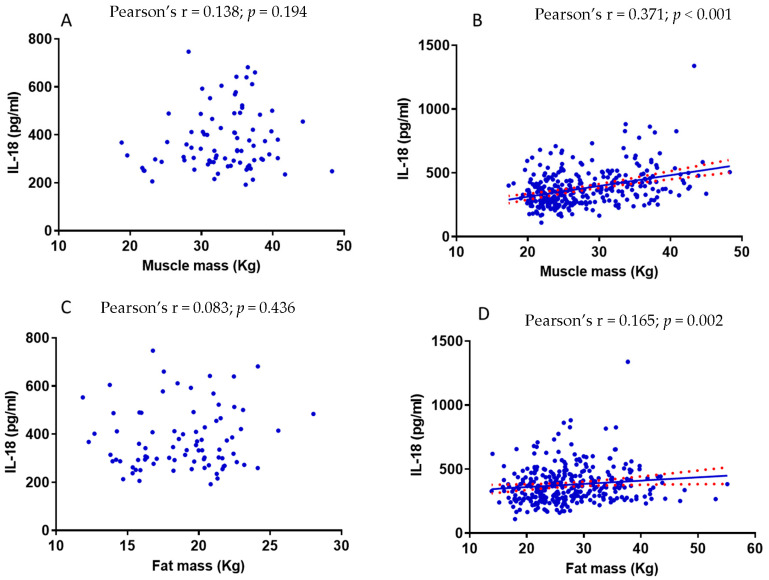
Pearson correlation analysis between IL-18 circulating levels and (**A**) muscle mass in subjects with fat mass < 30%; (**B**) muscle mass in subjects with fat mass ≥ 30%; (**C**) fat mass in subjects with fat mass < 30% and (**D**) fat mass in subjects with fat mass ≥ 30%. Data analysis was performed using log transformation. Pearson’s r, Pearson correlation coefficient.

In order to gather more insights into the relationship between muscle mass and IL-18 circulating levels, we performed a subgroup analysis dividing study participants according to their fat mass. In this regard, muscle mass did not correlate with IL-18 plasma levels in individuals with a fat mass < 30% (*p* = 0.194) (Figure 3A). However, this correlation became significant when considering study participants with a fat mass ≥ 30% (*p* < 0.001) (Figure 3B). The same was true when considering fat mass instead of muscle mass. Indeed, in individuals with a fat mass < 30%, the fat mass (expressed in Kg) did not correlate with IL-18 plasma levels (Figure 3C), whereas the opposite occurred in subjects with a fat mass ≥ 30% (Figure 3D).

## 4. Discussion

The data reported herein provide evidence on the role of dietary fatty acids on the regulation of IL-18 circulating levels and further support the tight relationship between this pro-inflammatory cytokine and insulin resistance in a cohort of non-diabetic individuals. Furthermore, the results of the present study highlight the role of dietary fatty acid quality, particularly the ratio between omega-3 and saturated fatty acids, as a moderate predictor of IL-18 circulating levels.

IL-18 has been previously reported to be upregulate in obese, insulin-resistant individuals, with this cytokine being tightly associated with insulin resistance [12,13,14,15]; this relationship was confirmed as part of this study. This association is not surprising, indeed metabolic inflammation, typically occurring in obese individuals [5,6,37,38], has been casually linked with insulin resistance. In support of this, infusion of the pro-inflammatory cytokine TNF-α in healthy humans has been shown to trigger insulin resistance [8,39]. Remarkably, the infusion of pro-inflammatory cytokines also led to an upregulation of IL-18 in skeletal muscle but not in adipose tissue [39]. This is in agreement with the present study, which identified muscle mass as a predictor or IL-18 circulating levels, further supporting the role of this tissue in contributing to the plasma levels of this cytokine, particularly in response to pro-inflammatory stimuli. This effect is further confirmed by the fact that muscle mass positively correlated with IL-18 plasma levels only in individuals with a fat mass higher than 30%, suggesting that adipose tissue overexpansion and possibly its release of pro-inflammatory cytokines triggers IL-18 secretion by the skeletal muscle [39]. In light of this, IL-18, alongside TNF-α, may be implicated in the pathogenesis of insulin resistance. However, direct evidence on the causal role of IL-18 in the pathogenesis of insulin resistance are still lacking. Despite this, an increase in the circulating levels of this cytokine has been shown to predict the onset of T2DM [40,41], which, independently of whether it may have a causal role in triggering insulin resistance, supports the role of IL-18 as a plausible predictor of insulin resistance. This possibility is also supported by the results of this study, which indicated that IL-18 increases the predictive power of waist circumference and circulating triglycerides for insulin resistance and that insulin-resistant individuals, as well as those affected by the metabolic syndrome, have higher IL-18 circulating levels compared with their respective counterparts (study participants with a HOMA-IR < 2.5 and those without a diagnosis of metabolic syndrome, respectively).

In terms of the role of diet, although the intake of the omega-3 fatty acids EPA and DHA and the ratios of monounsaturated/saturated fatty acids, polyunsaturated/saturated fatty acids and omega-3/saturated fatty acids correlated negatively with the circulating levels of IL-18, no relationship between total lipid intake and IL-18 plasma levels was identified. This is in line with the fact that dietary fatty acid quality, rather than quantity, is pivotal in shaping inflammation [42,43]. In this regard, while long-chain saturated fatty acids have been implicated in driving metabolic inflammation, the monounsaturated fatty acid oleic acids and the omega-3 fatty acids EPA and DHA were proven to be anti-inflammatory [10,30,36,44,45]. This paradigm is in line with the present data, which emphasise the quality of fatty acid intake as well as their ratios as a key factors in promoting inflammation. Indeed, although saturated fatty acids have been widely proposed to be pro-inflammatory, particularly in in vitro studies [10,25,46,47], herein, the intake of saturated fatty acids only tended to correlate with IL-18 plasma levels. On the contrary, this correlation become significant when considering the monounsaturated, omega-3 as well as polyunsaturated fatty acids/saturated fatty acids ratio, with the ratio between omega-3 and saturated fatty acids also being able to predict IL-18 circulating levels. To the same extent, despite EPA and DHA correlate negatively with IL-18 circulating levels. Nevertheless, none of them was able to independently predict IL-18 plasma levels, supporting the notion that their ratio with saturated fatty acids may be a more important discriminant in modulating metabolic inflammation. The findings of this study are in agreement with the notion that the impact of diet on inflammation is not dictated by isolated nutrients, instead, it is the direct consequence of the balance between the intake of pro- and anti-inflammatory nutrients, including fatty acids [42]. Not surprisingly, both oleic acid and omega-3 fatty acids have been reported to be able to rewire saturated fatty acid metabolism and therefore mitigate the pro-inflammatory and metabolically deleterious effects of palmitic acid. Indeed, oleic acid has been shown to channel palmitic acid towards the synthesis of triglycerides, thereby decreasing its bioavailability for the synthesis of lipotoxic metabolites, namely ceramides and diacylglycerols [48,49]. Additionally, oleic acid and EPA have been shown to increase fatty acid β-oxidation [50,51], which, in turn, may represent a further mechanism by which these unsaturated fatty acids decrease the pro-inflammatory effects of saturated fatty acids by decreasing their bioavailability. 

Nevertheless, despite saturated fatty acids, as opposed to monounsaturated fatty acids and polyunsaturated fatty acids, having been implicated in the pathogenesis of insulin resistance [10,30,52,53,54,55], in the present study the quality of fatty acids consumed was not identified as a predictor of insulin resistance. Despite this appearing counterintuitive, it is not entirely surprising. Indeed, rather than saturated fatty acids themselves, inflammation and lipotoxicity are pivotal in mediating the impact of these dietary fatty acids on insulin resistance [29,53,56,57,58]. In particular, this study provides further support to the pathophysiological relevance of inflammation in this process, highlighting the role of IL-18 as an inflammatory biomarker linking a shift in dietary lipid intake towards saturated fatty acid and insulin resistance.

This study presents some limitations in that it did not directly confirm the quality of the fatty acids consumed by its participants. Despite 24 h recalls being a validated method to assess nutrient intake, this method is not as accurate as the assessment of erythrocyte plasma membrane fatty acid composition to evaluate fatty acid intake. Another potential limitation of this study is that it is limited to individuals aged between 55–80 years, with the present findings being limited to this age group. Nevertheless, the data reported herein are of particular interest in order to evaluate the parameters that influence unhealthy aging. Finally, the sample size of the cohort evaluated as part of this study represent a strength, in association with the novelty of the findings reported herein. This is, in fact, the first report describing the relationship between dietary lipid intake, IL-18 circulating levels and insulin resistance.

In conclusion, the data reported herein support the importance of the balance between omega-3 and saturated fatty acids intake in predicting IL-18 circulating levels, which, in turn, may impact upon insulin sensitivity. Despite the quality of dietary lipids failing the predict insulin resistance, it was able to predict IL-18 plasma levels. This suggests the downregulation of IL-18 being key in underpinning, at least in part, the beneficial metabolic effects of substituting omega-3 for saturated fatty acids. Thus, this cytokine represents a potential biomarker bridging the gap between the quality of dietary fatty acids consumed, their ratio and insulin resistance.

## Figures and Tables

**Table 1:** Study participant characteristics.

Subjects, number	403
Female, number (%)	230 (57)
Age (years)	66 ± 5
Systolic blood pressure (mmHg)	138 ± 19
Diastolic blood pressure (mmHg)	85 ± 10
Body mass index (kg/m^2^)	26.5 ± 3.7
Waist circumference (cm)	92.1 ± 10.3
Fat-free mass (%)	64.6 ± 6.6
Fat-free mass (kg)	46.2 ± 9.3
Fat mass (%)	35.4 ± 6.6
Fat mass (kg)	25.5 ± 7.2
Muscle mass (kg)	28.8 ± 6.5
Glucose (mg/dL)	96.3 ± 10.6
Insulin (U/L)	9.0 ± 5.0
HOMA-IR	2.2 ± 1.4
Total cholesterol (mg/dL)	218.8 ± 37.7
HDL cholesterol (mg/dL)	67.6 ± 17.3
LDL cholesterol (mg/dL)	131.6 ± 33.0
Triglycerides (mg/dL)	98.5 ± 42.6
hsCPR (mg/dL)	0.207 ± 0.306
IL-18 (pg/mL)	377.6 ± 139.1
MetS, number (%)	49 (12.2)
Subjects therapy:	
Antihypertensive drugs, number (%)	109 (27)
Beta blockers, number (%)	24 (6)
Hypolipidemic therapy, number (%)	64 (15.9)

Data are expressed as mean ± SD or as number (%) or N (%). SD, standard deviation; HOMA-IR, homeostatic model assessment for insulin resistance; HDL, high density Lipoprotein; LDL, low density lipoprotein; hsCPR, high-sensitivity *C*-reactive protein; IL-18, interleukin 18; MetS, metabolic syndrome.

**Table 2:** Spearman correlation between Il-18 and HOMA-IR with parameters of interest.

	HOMA-IR		IL-18	
	rho	*p* Value	rho	*p* Value
Age (years)	0.048	0.346	0.025	0.617
Systolic blood pressure (mmHg)	0.148	0.003	0.086	0.084
Diastolic blood pressure (mmHg)	0.130	0.010	0.136	0.006
Body mass index (kg/mq)	0.515	<0.001	0.176	<0.001
Waist circumference (cm)	0.503	<0.001	0.232	<0.001
Fat-free mass (%)	−0.224	<0.001	0.085	0.089
Fat-free mass (Kg)	0.287	<0.001	0.266	<0.001
Fat mass (%)	0.224	<0.001	−0.085	0.089
Fat mass (Kg)	0.458	<0.001	0.124	0.013
Muscle mass (Kg)	0.304	<0.001	0.292	<0.001
Glucose (mg/dL)	0.529	<0.001	0.180	<0.001
Insulin (U/L)	0.979	<0.001	0.234	<0.001
HOMA-IR	-	-	0.247	<0.001
Total cholesterol (mg/dL)	−0.081	0.106	−0.138	0.006
LDL cholesterol (mg/dL)	−0.033	0.514	−0.066	0.188
HDL cholesterol (mg/dL)	−0.266	<0.001	−0.217	<0.001
Triglycerides (mg/dL)	0.320	<0.001	0.104	0.039
IL-18 (pg/mL)	0.247	<0.001	-	-
hsCRP (mg/L)	0.206	<0.001	0.202	<0.001

rho, Spearman ‘s rho coefficient; HOMA-IR, homeostatic model assessment for insulin resistance; HDL, high density lipoprotein; LDL, low density lipoprotein; IL-18, interleukin 18; hsCRP, high-sensitivity *C*-reactive protein.

**Table 3:** Spearman’s rho correlation between Il-18 and dietary parameters.

	HOMA-IR		IL-18	
	rho	*p* Value	rho	*p* Value
Total calories (kcal/day)	−0.003	0.958	0.083	0.097
Alcohol (kcal/day)	0.018	0.724	0.037	0.462
Protein (g/day)	−0.005	0.917	0.007	0.889
Lipid (g/day)	0.034	0.496	0.031	0.541
Available carbohydrates (g/day)	−0.044	0.380	0.071	0.154
Starch (g/day)	0.070	0.166	0.061	0.218
Total fibre (g/day)	−0.153	0.002	−0.066	0.186
Cholesterol (mg/day)	0.114	0.023	0.051	0.311
SFA (g/day)	0.068	0.174	0.091	0.068
MUFA (g/day)	−0.048	0.336	−0.029	0.556
PUFA (g/day)	−0.019	0.704	−0.015	0.760
MUFA/SFA ratio	−0.163	0.001	−0.167	0.001
PUFA/SFA ratio	−0.092	0.068	−0.142	0.004
(MUFA + PUFA)/SFA ratio	−0.166	0.001	−0.169	<0.001
C20:5 EPA (g/day)	−0.094	0.061	−0.100	0.045
C22:6 DHA (g/day)	−0.128	0.011	−0.137	0.006
Omega-3/SFA ratio	−0.166	0.001	−0.204	<0.001
Omega-6/Omega-3 ratio	0.129	0.011	0.095	0.057
Mediterranean diet adherence	−0.084	0.094	−0.101	0.044

rho, Spearman’s rho correlation coefficient; SFA, saturated fatty acids; MUFA, monounsaturated fatty acids; PUFA, polyunsaturated fatty acids; EPA, eicosapentaenoic acids; DHA, docosahexaenoic acid.

**Table 4:** Multiple linear regression model indicating predictors of HOMA-IR.

Model	R^2^	*p* Value Model	Predictor	Unstandardized B Coefficient	*p* Value
1	0.257	<0.001	Waist circumference (cm)	0.011	<0.001
2	0.311	<0.001	Waist circumference (cm)	0.010	<0.001
			Triglycerides (mg/dL)	0.270	0.001
3	0.334	<0.001	Waist circumference (cm)	0.005	0.001
			Triglycerides (mg/dL)	0.285	0.002
			IL-18 (pg/mL)	0.017	0.031

**Model 1** Adjusted for SBP (mmHg); DBP (mmHg); Fat mass (Kg); Muscle mass (Kg); Total cholesterol (mg/dL); HDL cholesterol (mg/dL); Triglycerides (mg/dL); IL-18 (pg/mL); MUFA/SFA ratio; PUFA/SFA ratio; (MUFA + PUFA)/SFA ratio; C20:5 EPA (g/day); C22:6 DHA (g/day); Omega-3/SFA ratio; Omega-6/Omega-3 ratio; Mediterranean diet adherence. **Model 2** Adjusted for SBP (mmHg); DBP (mmHg); Fat mass (Kg); Muscle mass (Kg); Total cholesterol (mg/dL); HDL cholesterol (mg/dL); IL-18 (pg/mL); MUFA/SFA ratio; PUFA/SFA ratio; (MUFA + PUFA)/SFA ratio; C20:5 EPA (g/day); C22:6 DHA (g/day); Omega-3/SFA ratio; Omega-6/Omega-3 ratio; Mediterranean diet adherence. **Model 3** Adjusted for SBP (mmHg); DBP (mmHg); Fat mass (Kg); Muscle mass (Kg); Total cholesterol (mg/dL); HDL cholesterol (mg/dL); MUFA/SFA ratio; PUFA/SFA ratio; (MUFA + PUFA)/SFA ratio; C20:5 EPA (g/day); C22:6 DHA (g/day); Omega-3/SFA ratio; Omega-6/Omega-3 ratio; Mediterranean diet adherence. HOMA-IR, homeostatic model assessment for insulin resistance; SBP, systolic blood pressure; DBP, diastolic blood pressure; HDL, high density lipoprotein; IL-18, interleukin 18; MUFA, monounsaturated fatty acids; SFA, saturated fatty acids; PUFA, polyunsaturated fatty acids; EPA, eicosapentaenoic acids; DHA, docosahexaenoic acid. Abnormal distribution variables entered into the model after log transformation (Fat mass, Muscle mass, HOMA-IR, Triglycerides, IL-18, MUFA/SFA ratio, PUFA/SFA ratio, (MUFA + PUFA)/SFA ratio, C20:5 EPA; C22:6 DHA, Omega-3/SFA ratio, Omega-6/Omega-3 ratio).

**Table 5:** Multiple linear regression model indicating predictors of IL-18.

Model	R^2^	*p* Value Model	Predictor	Unstandardized B Coefficient	*p* Value
1	0.126	<0.001	Muscle Mass (kg)	0.007	<0.001
2	0.182	<0.001	Muscle Mass (kg)	0.007	<0.001
			Omega-3/SFA Ratio	−0.073	0.003
3	0.211	<0.001	Muscle Mass (kg)	0.005	0.006
			Omega-3/SFA Ratio	0.285	0.002
			HDL Cholesterol (mg/dL)	0.017	0.027
4	0.239	<0.001	Muscle Mass (kg)	0.006	0.001
			Omega-3/SFA Ratio	−0.069	0.001
			Cholesterol HDL (mg/dL)	−0.001	0.011
			Total Cholesterol (mg/dL)	−0.027	0.027

**Model 1** Adjusted for DBP (mmHg); Waist circumference (cm); Fat mass (Kg); HOMA-IR; Total cholesterol (mg/dL); HDL cholesterol (mg/dL); Triglycerides (mg/dL); MUFA/SFA ratio; (MUFA + PUFA)/SFA ratio; C20:5 EPA (g/day); C22:6 DHA (g/day); Omega-3/SFA ratio; Omega-6/Omega-3 ratio; Mediterranean diet adherence; Antihypertensive drugs (including beta-blockers); Beta-blockers; Hypolipidemic therapy. **Model 2** Adjusted for DBP (mmHg); Waist circumference (cm); Fat mass (Kg); HOMA-IR; Total cholesterol (mg/dL); HDL cholesterol (mg/dL); Triglycerides (mg/dL); MUFA/SFA ratio; (MUFA + PUFA)/SFA ratio; C20:5 EPA (g/day); C22:6 DHA (g/day); Omega-6/Omega-3 ratio; Mediterranean diet adherence; Antihypertensive drugs (including beta-blockers); Beta-blockers; Hypolipidemic therapy. **Model 3** Adjusted for DBP (mmHg); Waist circumference (cm); Fat mass (Kg); HOMA-IR; Total cholesterol (mg/dL); Triglycerides (mg/dL); MUFA/SFA ratio; (MUFA + PUFA)/SFA ratio; C20:5 EPA (g/day); C22:6 DHA (g/day); Omega-6/Omega-3 ratio; Mediterranean diet adherence; Antihypertensive drugs (including beta-blockers); Beta-blockers; Hypolipidemic therapy. **Model 4** Adjusted for DBP (mmHg); Waist circumference (cm); Fat mass (Kg); HOMA-IR; Total cholesterol (mg/dL); Triglycerides (mg/dL); MUFA/SFA ratio; (MUFA + PUFA)/SFA ratio; C20:5 EPA (g/day); C22:6 DHA (g/day); Omega-6/Omega-3 ratio; Mediterranean diet adherence; Antihypertensive drugs (including beta-blockers); Beta-blockers; Hypolipidemic therapy. IL-18, interleukin 18; BMI, body mass index; HOMA-IR, homeostatic model assessment for insulin resistance; HDL, high density lipoprotein; MUFA, monounsaturated fatty acids; SFA, saturated fatty acids; PUFA, polyunsaturated fatty acids; EPA, eicosapentaenoic acids; DHA, docosahexaenoic acid. Abnormal distribution variables entered into the model after log transformation (Fat mass, Muscle mass, HOMA-IR, Triglycerides, IL-18, MUFA/SFA ratio, PUFA/SFA ratio, (MUFA + PUFA)/SFA ratio, C20:5 EPA; C22:6 DHA, Omega-3/SFA ratio, Omega-6/Omega-3 ratio).

## Data Availability

The data supporting the study findings are available on request from the corresponding author [A.P.]. Data are not publicly available due to the PANGeA study consortium agreement, which regulates the intellectual property of the data.
